# Mammalian dental diversity: an evolutionary template for regenerative dentistry

**DOI:** 10.3389/fdmed.2023.1158482

**Published:** 2023-04-26

**Authors:** Tracy Popowics, Priti Mulimani

**Affiliations:** Department of Oral Health Sciences, University of Washington School of Dentistry, Seattle, WA, United States

**Keywords:** tooth morphology, mammals, odontogenesis, diversity, dental function, evolution

## Abstract

The discovery of odontogenic mechanisms essential for regenerating dental tissues and eventually developing a biomimetic artificial whole tooth for replacement is an ongoing aspiration for dental clinicians and researchers. Studying the diversity, development and evolutionary changes of mammalian dentitions can provide key insights into the mechanisms of odontogenesis that can be harnessed for regenerative dental medicine. A myriad of influences is expected to have shaped the dentitions of mammals and our objective is to highlight the contributions of phylogeny, functional adaptation, and development to tooth shape. Innovations in tooth shape analysis will be discussed, such as in imaging methodologies and quantitative comparisons, molecular biology approaches to phylogeny and the ontogenetic basis of tooth form. Study of the inter- and intra-species differences in tooth form as well as dental anomalies has provided clues toward the mechanisms of evolutionary change in dental form. Thus, phenotypic variation in tooth shape will also be discussed, including the role of development in creating tooth shape differences that evolutionary selection pressures may act upon. Functional adaptations have occurred in the context of the phylogenetic signal of primitive mammals, and predecessors to each phylogenetic branch, and examples will be discussed within members of the Order Carnivora, the Superfamily Suoidea and the Order Primates. The comparative study of mammalian tooth shape holds the potential to inform dental research areas, such as etiopathogeneses of dental variation and tooth shape anomalies, molecular mechanisms of tooth development and functional issues. Ultimately, insights from these research areas can be potentially translated for futuristic clinical applications like regeneration of various tooth tissue layers and eventually full tooth replacement.

## 1. Introduction

The Class Mammalia has approximately 5,500 species in 29 Orders, and its members are characterized by having mammary glands which in females produce milk for feeding their young, a neocortex, fur or hair and three middle ear bones. Mammals are broadly classified into two subclasses, Prototheria (egg-laying mammals, e.g., echidna and platypus) and Theria (live-bearing mammals). The Theria are classified further into (1) Metatheria (marsupials), consisting of pouched mammals like kangaroos, wombats, opossums, etc. whose newborns are altricial and need to be carried and suckled in the mother's external pouch to develop fully and (2) Eutheria (placental mammals), a group that gives live birth to precocial young and includes Orders like Rodentia (rodents), Chiroptera (bats), Artiodactyla (pigs, cattle, giraffes, camels, sheep, goats), Cetacea (whales, dolphins, porpoises), Carnivora (cats, dogs, weasels, bears) and Primates (humans, apes, monkeys, lemurs) (1). Mammalian teeth display great diversity in terms of their shapes, sizes, numbers, function, and eruption patterns. The unique combinations, made possible by various elements of the dentition, give rise to distinct species-specific characteristics. As early as 350 B.C., Aristotle's observations on tooth number, shape and eruption introduced the comparative study of mammalian teeth (2). In more recent times, the specificity of these dental traits in each organism has enabled identification of living mammalian species, as well as those in the fossil record. Hence, distinguishing tooth characteristics have been valuable in charting the evolutionary history of a species and their inter relationships through the construction of phylogenies. Given this species-specific regularity of tooth shape, many authors have puzzled over how evolutionary change occurs in association with variation in dental morphology (3–5). This leads to the questions: (1) what type of variability regularly occurs in mammalian dentitions, and (2) have the mechanisms responsible for this variability been co-opted by the evolutionary process to generate dental diversity? To answer these questions, this review will discuss key determinants of mammalian tooth shape and the variation that exists within the toothrow. Furthermore, examples of how these determinants have shaped taxonomic differences in the mammalian dentition will also be presented. These examples are limited to taxa with low-crowned (bunodont) teeth, excluding high-crowned (hypsodont) teeth, because of the greater similarity of low-crowned mammalian teeth to the human dentition. New approaches for understanding the relationship between dental development, morphological variation, and dental evolution will also be considered, as well as the relevance of these data to clinical treatment and emerging dental therapies.

## 2. Determinants of dental diversity

More complete discussions of mammalian dental diversity can be found within Teaford et al. (6) and Ungar (7). Here the contributions of phylogenetic signal, mechanical function, and dental development to species-specific differences are considered.

### 2.1. Phylogenetic signal

The phylogenetic signal is an evolutionary term that refers to the tendency of related biological species to resemble each other more than they resemble species drawn at random from a phylogenetic tree, i.e., similarity of close relatives compared to distant relatives. Phylogenetic signal in dental morphology corresponds with the dental features that have been inherited from a common ancestor and shared among closely related taxa. The phylogenetic signal has great depth among vertebrates, with homologous cusps extending from reptiles to early synapsids (stem mammals). Mammalian teeth have evolved from an ancestral conical shape, or one-cusped, tooth to progressively more elaborate shapes (7, 8). Phylogenetic signals that are carried throughout the mammalian radiation include homology with the tribosphenic tooth shape and heterodonty, as will be discussed below.

**Figure 1:**
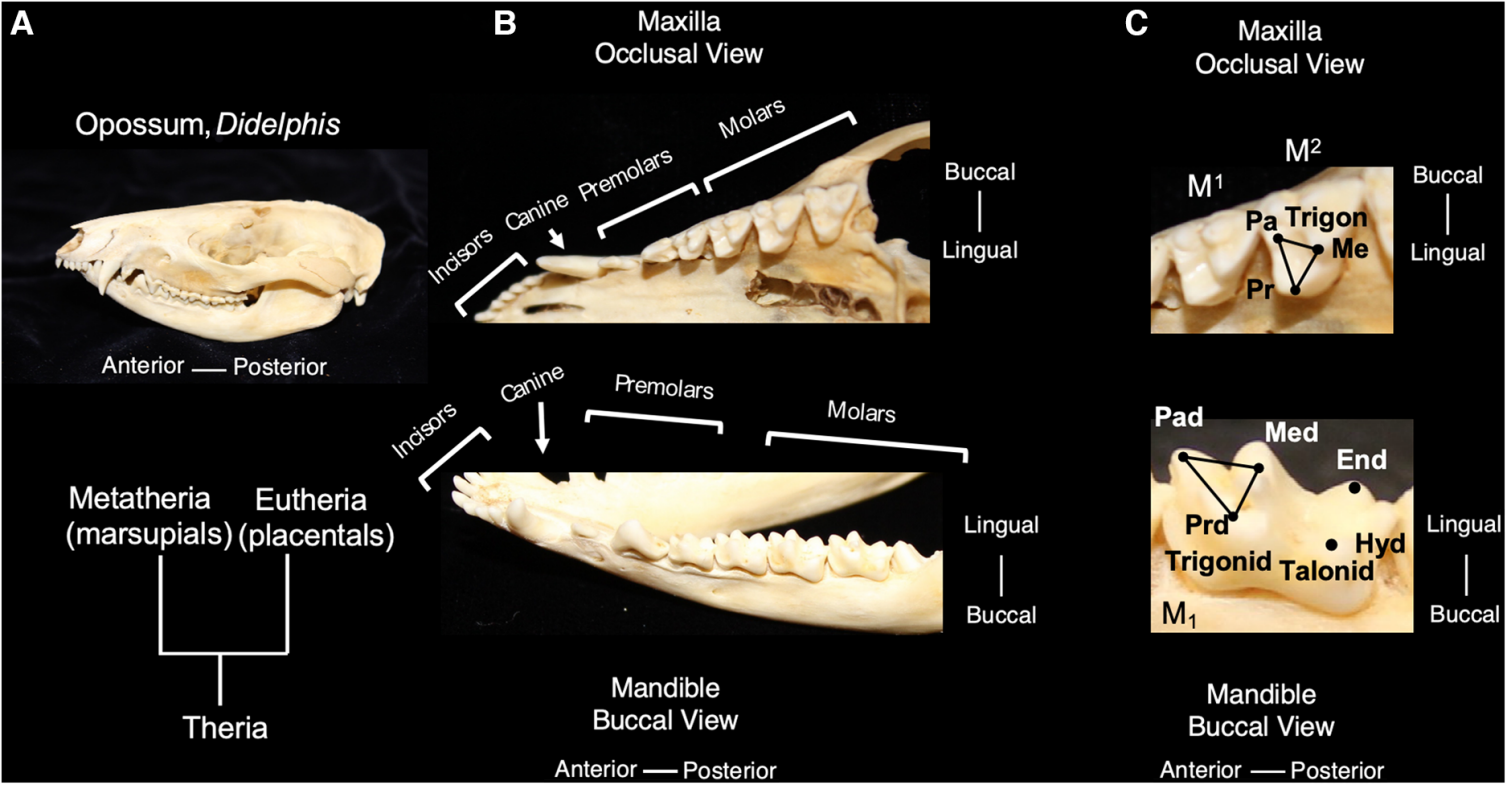
The tribosphenic molar shape is present in advanced therian mammals, the common ancestor to metatherian and eutherian mammals; molars of the opossum, *Didelphis virginianus*, are representative of this primitive tooth shape (**A**). An occlusal view of the maxilla and buccal view of the mandible show the tooth classes present in *Didelphis* (**B**). The trigon in the maxillary molars of *Didelphis* includes the tooth cusps of paracone (pa) metacone (me) and protocone (pr) and is highlighted with black lines. The trigonid in the mandibular molars includes the tooth cusps of paraconid (pad), metaconid (med) and protoconid (prd) and is highlighted with black lines. The talonid is the region posterior of the trigonid and includes the posterior cusps, hypoconid (hyd) and entoconid. (**C**) Teeth are not shown to scale.

**Figure 2:**
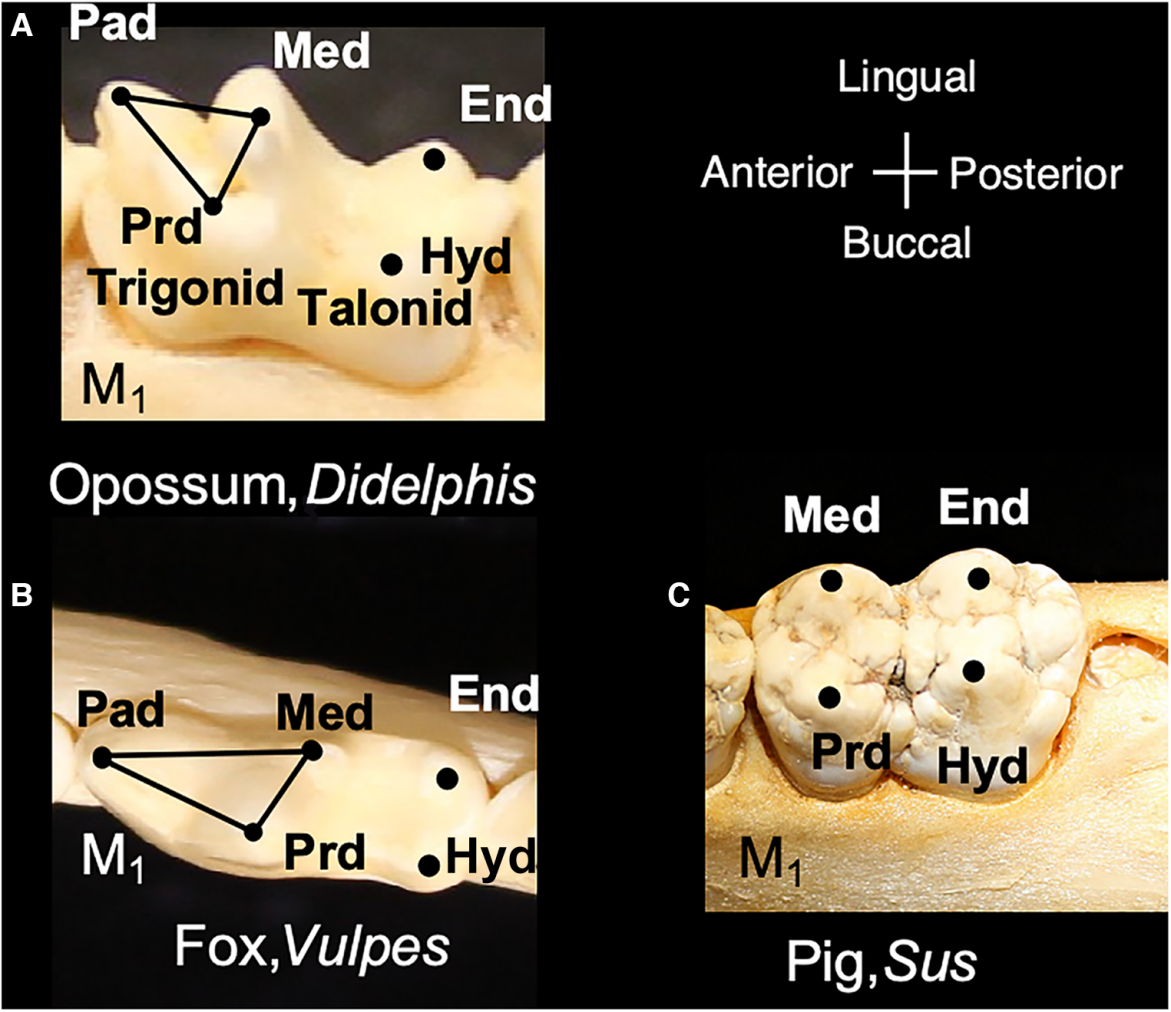
Modern mammals include molar shapes that have evolved from the tribosphenic shape. (**A**) Lower molar of *Didephis*, showing the location of the trigonid and talonid cusps. (**B**) Lower molar of the fox, *Vulpes*, showing emphasis of the paraconid (pad) and protoconid (prd) into a carnassial blade and reduction of the metaconid (med). (**C**) Lower molar of the pig, *Sus*, showing the absence of the paraconid and emphasis of the remaining trigonid cusps (med and prd) and talonid cusps (hypoconid, hyd, and entoconid, end). Teeth are not shown to scale.

Living mammals share tooth cusp homology with a primitive molar pattern, known as the “tribosphenic molar,” present in the common ancestor to metatherian (marsupial) and eutherian (placental) mammals (8). The tribosphenic molar appeared in the Late Jurassic 160 mya. and emerged following the appearance of multiple intermediary shapes, such as three cusps positioned in a straight line and the later arrangement of cusps in a triangle in early mammals. A tribosphenic molar is one that is capable of both grinding (tribein) and shearing (sphen) occlusal functions by virtue of having a triangular 3-cusp pattern wherein the lingual cusp of the upper molar occludes within a distal basin on the lower molars (9) (Figure 1). The triangular area that includes a lingual cusp called protocone, a mesiobuccal cusp called paracone and a distobuccal cusp called metacone in the upper molar is referred to as a trigon. Of these, the paracone is the oldest evolutionarily and shares homology with the central cusp of triconodont molars in early mammals. The same structures are present in lower molars but have the suffix -id attached to their names. Additionally, the protoconid is buccal whereas the paraconid and metaconid are on the lingual side. Additionally, lower molars have a basin distal to the trigonid called a talonid or the talonid basin which is ringed by three cusps, the buccal hypoconid, lingual entoconid, and between them, the hypoconulid function (10, 11). The cusps on anterior teeth, such as premolars, also share homology with the tribosphenic pattern. Among extant mammals, the opossum, *Didelphis virginianus*, most closely represents this primitive condition for molar shape, including the trigon and trigonid structure of primitive therians. This primitive tooth shape is presumed to be the prototype from which all other mammalian dentitions are derived; thus, fossil and modern mammals share cuspal homology with this primitive state. The transformation from this primitive pattern to more derived tooth shapes is a key aspect of the Cope-Osborn theory (8, 12). The cusp homology maintained between the primitive and derived dental shapes thus constitutes a phylogenetic signal within the dentition (Figure 2).

In mammalian heterodonty, the division of the dentition into tooth classes is also a phylogenetic signal shared among mammals. Mammals inherited the presence of tooth classes from nonmammalian cynodont (more advanced synapsids) ancestors (11). Although most species retain this feature to some degree, heterodonty has been secondarily lost in others, such as dolphins and other toothed cetaceans (7, 13). The dentition of primitive living placentals includes 3 incisors, 1 canine, 4 premolars and 3 molars in the upper and lower toothrows. For marsupials, *Didelphis* contains the primitive number of teeth in each tooth class with 5 incisors, 1 canine, 3 premolars and 4 molars in the upper toothrow and a similar number in the lower toothrow, except for only 4 incisors (Figure 1). Evolutionary changes to tooth number have occurred in numerous mammalian lineages and often correspond with dietary specialization (3).

Multiple statistical analyses have been used to tease apart the relative contribution of phylogeny to tooth shape differences. These approaches assess the tendency for related species to resemble one another more than a randomly selected species from a phylogenetic tree (14–16). The species-specific nature of the dentition, as well as the prevalence of teeth in the fossil record, has favored the use of dental characters in mammalian phylogenetics. Thus, the extent to which a species shares a dental feature has often been used to determine relatedness and has a potentially confounding effect. Many studies bypass this issue through comparison of tooth morphology with phylogenetic assessments based on non-dental characters, including mitochondrial and nuclear sequences from extant species (17–20). Application of such methods to the dentitions of species in didelphids (opossum family) found phylogeny to explain the majority of tooth shape differences relative to other factors such as size and habitat (21). Likewise, the assessment of the phylogenetic signal in carnivoran dentitions demonstrated the significant role of phylogeny in determining the relationship between tooth shape and diet (22). In bats, phylogeny was also found to be the greatest predictor of anomalies in tooth number (17). In contrast, molar shape variation in wild murines (mice) and mouse mutants showed surprisingly low phylogenetic signal. This suggests convergent evolution in which distantly related organisms independently evolve similar traits as a result of having to adapt to similar environments or ecological niches. In murine dentitions common diets are likely to have led to similar dental adaptations (20).

### 2.2. Mechanical function

The primitive mammalian tribosphenic molar's shearing and crushing functions are the basis for the evolution of more derived shapes and functions. The high metabolic rate of mammals relative to their predecessors corresponds with increased pressure to feed efficiently (23, 24). The evolution of tooth shape diversity is largely considered to correspond with the necessity for a high caloric intake (7, 11). Mechanical breakdown of food in the mouth prior to swallowing improved access to new dietary niches, contributing to mammalian evolutionary success. The types of foods ingested and the mechanical properties of the diet provided selection pressure towards matching an appropriate tooth shape to the food ingested (25, 26). Chewing breaks ingested foods into smaller pieces, thus increasing the surface area of the foods for the activity of digestive enzymes, both in the oral cavity and gut (27, 28). Without the masticatory tools to breakdown ingested food, the foodstuff may pass through the digestive tract undigested. Foods with high toughness can be efficiently reduced with opposing blades on occluding teeth. Rather than loading the bladed surfaces all at once, the opposing blades form point contacts and concentrate bite forces on a small area within the foodstuff. This method is well-suited to initiating a tear in and fracturing tough vertebrate or plant material (25, 29). In contrast, more broadly curved cusps function well in fracturing hard foods (30). Hard foods, such as some seeds or insects, can be trapped between a rounded cusp and a basined surface on the occluding tooth. As the blunt cusp concentrates stress on the hard food, cracks occur in the food without breakage to the cusp itself. The effectiveness of the tribosphenic molar shape in including both shearing and crushing surfaces is evident from the retention of this form in numerous mammal groups, such as opossums and bats. Other mammalian molar shapes include modification of the size and shape of the tribosphenic pattern to further emphasize the shearing or crushing function of the dentition, as will be discussed in Section 2.4.

The number of teeth present in each tooth class varies among mammals and is not only important as a taxonomic consideration but also as a functional adaptation. Incisors function in food intake and grooming, whereas canines pierce or stab food and/or contribute to aggressive displays. The premolars and molars mechanically process the food prior to swallowing. Depending on the shape and orientation of the chewing cycle (jaw movement), different regions of the tooth row may experience different bite forces (31). The location of maximum bite force along the toothrow can correspond with tooth shape changes that maximize the applied force. For example, both bats and carnivorans show an orthal (scissor-like) chewing stroke and bite forces corresponding with evolutionary specializations of teeth. In general, the muscles of mastication produce bite forces in bats that decrease from the incisors to the canines; however, at the premolars, bite forces increase and continue to do so through the molars. More insectivorous species show higher bite force more anteriorly in the toothrow whereas frugivores/omnivores show higher bite forces posteriorly (32). Members of the Order Carnivora all share the loss of the third upper molar (M^3^) and further reduction of the toothrow is associated with reduction of premolars and molars in different families (dog, cat, weasel, bear and the like) (33). The focused bite forces on the remaining premolars and molars correspond with morphological diversification related to dietary preferences ranging from carnivory/omnivory to bone or shell-crushing (34, 35).

Comparing tooth shape with function across different mammalian radiations includes both traditional assessments of functional characteristics and more data-intensive approaches. Historically, study of tooth functional adaptations has used linear measurements of dental features that rely on identification of landmarks across specimens. These methods are still in use and facilitate comparisons between different data sets (22, 34, 36). Another approach has been to identify homologous landmarks on comparable teeth and to superimpose these landmarks on a grid to create partial warp (PW) scores. The PW scores are multivariate shape descriptors that are calculated based on the difference between a specimen's and a consensus shape. In addition to quantifying the shape differences, this method permits visualization of the deformation of a grid when compared to the undeformed grid of the consensus shape (37). These approaches, however, can limit the study of occlusal shapes that lack landmarks or are modified through wear. With the advancement of imaging methodologies and the increased capacity to manage and store large data sets, newer methods are able to analyze more of the crown shape, as reviewed in the context of primates (38). Dental topographic analysis has gained prominence as a tool to understand the relationship between tooth shapes and diet (39, 40). The method does not require the use of landmarks but instead uses standard geographic information systems (GIS) measures that capture the occlusal relief and sloping angular surfaces. In an alternative approach, the orientation patch count rotated (OPCR) method partitions the crown surface into patches and contiguous areas of the crown with similar aspects and has been effective in analyses of tooth complexity (41).

### 2.3. Dental development

Tooth development occurs through the reciprocal and sequential induction of epithelial and neural crest-derived ectomesenchyme in the primordial first branchial/pharyngeal arch. Homeobox genes, containing a highly conserved DNA sequence of approximately 180 base pairs, play a role in coding for transcription factors that contribute to tooth row dental patterns. When neural crest cells arrive in the 1st arch, they bring with them the potential to express the homeobox-containing genes Barx, Dlx, Lhx, Pitx and Msx and are developmentally plastic without a predetermined fate. Signals from the oral epithelium (ectoderm) induce the expression of specific combinations of transcription factors in the ectomesenchyme that lead to specification of tooth shape (42, 43). This is the basis of the “odontogenetic homeobox code model” that posits that regional variation in homeobox transcription factors within the neural crest-derived ectomesenchyme along the jaw corresponds with development of different tooth classes (44). Continued signaling between the ectoderm and ectomesenchyme regulates the regional expression of transcription factors and advances tooth shape formation and cellular differentiation (45, 46).

**Figure 3:**
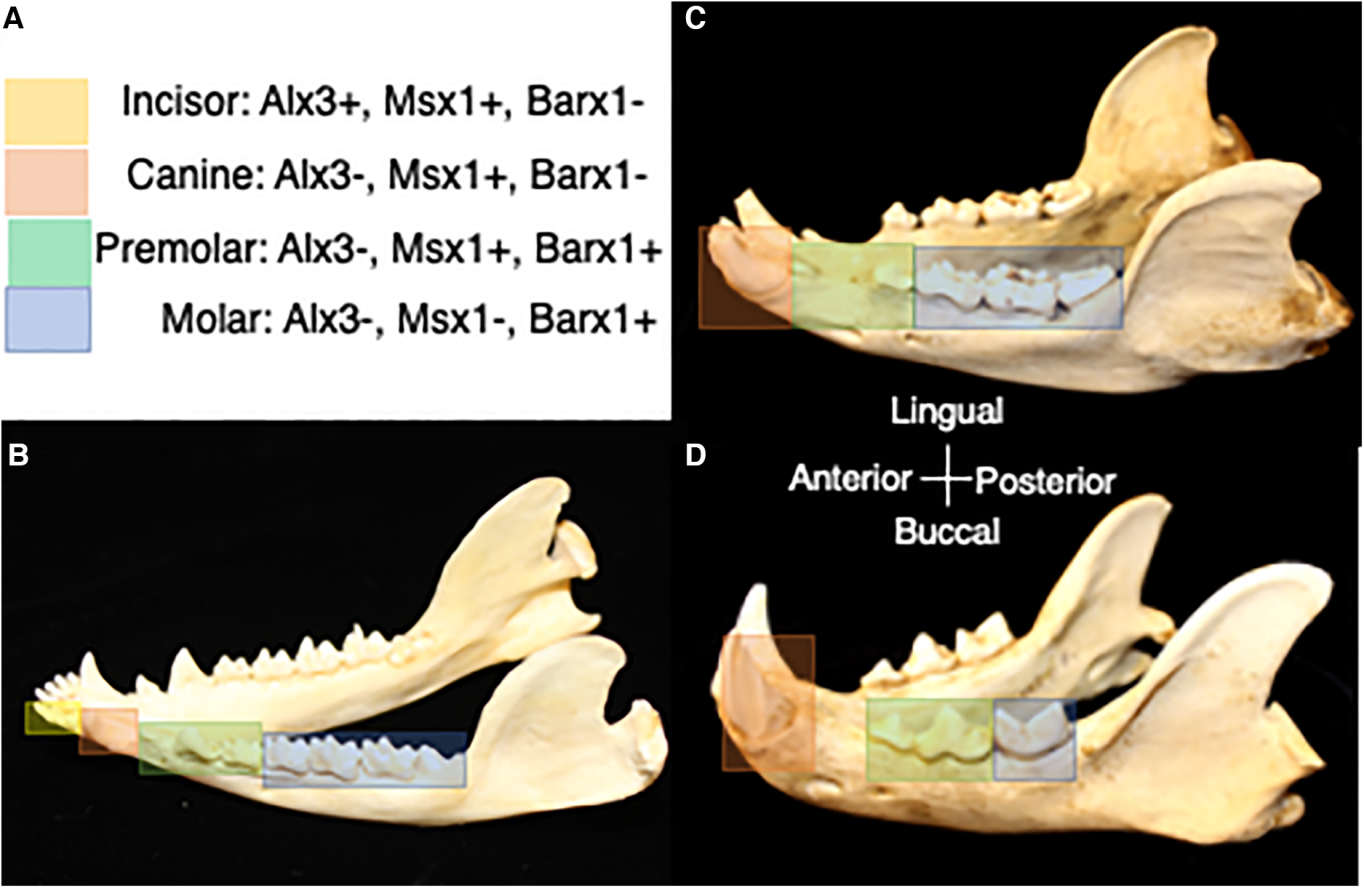
(**A**) Homeobox code expression in association with tooth classes in the prototypical mammal jaw (50). Yellow corresponds with expression of homeobox genes in the incisor region, orange corresponds with expression of homeobox genes in the canine region, green corresponds with expression of homeobox genes in the premolar region, and blue corresponds with expression of homeobox genes in the molar region. (**B**) Homeobox gene expression in association with tooth classes in the jaw of the opossum, *Didelphis*. (**C**) Predicted homeobox gene expression patterns in association with tooth classes in the jaw of the bear, *Ursus*. (**D**) Predicted homeobox gene expression patterns in association with tooth classes in the jaw of the cat, *Felis*. Differences in the range of expression of combinations of homeobox genes is expected to correspond with evolutionary changes in tooth class number. Dentitions are not shown to scale.

Understanding the role of homeobox-containing genes in tooth class specification has advanced our conceptualization of the evolution of heterodonty and tooth shape variation. Prior to the development of molecular biology techniques, Butler (1939) introduced the concept that “fields of influence” governed the development of mammalian tooth classes (47). In this model, teeth develop along the toothrow with an identical potential for shape formation and different tooth shapes are generated according to a gradient of environmental factors. Later, Osborn (1973) proposed that a single clone of preprogrammed mesenchymal cells was responsible for each of the teeth in a toothrow. In this “clone model,” mesenchymal replication generated individual teeth in sequence from the same clone and changes in the growth capacity of the replicating cells led to different tooth forms (48). The odontogenetic homeobox code model resonates with the “fields of influence” model in that ectoderm's induction of different combinations of genes within a specific region define the developmental field for incisors, canines, premolars and molars (42). A synthesis of these models is also possible with tooth shape reliant on the “clones” of neural crest-derived ectomesenchyme, the homeobox gene expression within the ectomesenchyme and the ectodermal signaling that elicits gene expression (49). Comparison of tooth development among mice, ferrets and opossums shows similar homeobox code expression for all four tooth classes, suggesting the presence of this gene expression pattern in the common ancestor to marsupial and placental mammals (50). Changes in the expression pattern of these genes have been implicated in the evolutionary changes in tooth class number within the mammalian dentition (Figure 3) (51). Mutations in homeobox genes have also been hypothesized to permit the expression of cryptic genetic differences that are usually not apparent in the phenotype contributing to variation in dental patterning (3).

Tooth shape also depends on the development of signaling centers, known as primary and secondary enamel knots, within the ectoderm-derived dental tissue. The primary enamel knot is the first signaling center to appear and initiates secondary knots within the epithelium. The sequential appearance of knots marks the location of sites of cusp formation. Epithelial growth is inhibited by key molecules at enamel knot sites but continues between the forming cusps. The timing and extent of this differential growth creates a three-dimensional shape within the epithelium that precedes the final tooth shape. Enamel knot signaling regulates the growth and differentiation of the dental tissues that secrete dentin and enamel and tooth shape through determination of cusp height and the order of dentin and enamel mineralization (52). The observation of enamel knot-like signaling centers in the development of shark teeth has led to the idea that enamel knot signaling centers are conserved among vertebrates and pre-date the evolution of teeth. The earliest enamel knot-like signaling center is proposed to have occurred outside of the oral cavity, in the development of dermal tooth-like structures and/or other epithelial appendages (53). Indeed, the same signaling pathways are found in cusp formation of fish, reptiles and mammals (45).

Computational models have provided examples of how enamel knot signaling, and other cellular processes may regulate tooth shape. For example, one model demonstrates the potential to generate diverse tooth cusp patterns through regulation of cusp formation with activator-inhibitor feedback loops. In this model, tooth shape results from successive appearance of enamel knots combined with tooth growth (54). Modification of this model demonstrates that increases in the activator component of the feedback loop could produce more tooth cusps and lead to a rectangular molar cusp pattern that has evolved in parallel within multiple mammalian lineages (55). Computer modeling has also been used to test the hypothesis that mechanical resistance to growth of the epithelial tissue in specific directions could account for the shape changes in the inner enamel epithelium that precede hard tissue deposition (56).

Clearly, gene mutations that lead to modification of signaling within the enamel knot and underlying mesenchyme have the potential to produce evolutionary change in molar form. Numerous mutations that cause a modification in cusp number have been observed, as reviewed in (45). In a comparative study of 236 tooth-associated genes analyzed in 39 mammalian genomes, positive selection signatures were identified in 31 genes. Older genes shared among vertebrates were less diverse than younger genes specific to mammals that showed higher evolutionary rates of change. Although the link between genes and tooth shape is ill-defined, these positively selected genes may be candidates responsible for mammalian tooth shape diversification (4). For example, mutations in the Fgf3 gene are present in both mice and humans and deficiency in Fgf3 signaling has been shown to lead to the reappearance of more ancestral molar phenotypes, i.e., reduction in cusp number (57). In contrast, increases in *Eda*, a signaling molecule also expressed in enamel knots, has been found to increase cusp formation (58).

Transcriptomic analysis of developing mouse teeth has led to the identification of genes regulating tooth development. Interestingly, these genes are rarely co-expressed and do not occur in the same gene neighborhood. Application of an algorithm (DeLocal) to detect small changes in gene expression among neighboring genes has allowed even subtle changes in gene expression that advance tooth development to be identified (59). Combining phenotypic information from mouse null mutations with single-cell level transcriptomic data has enabled the genes required for mouse tooth development to be identified. These are classified according to mechanistic category, i.e., enabling developmental progression, involved in tooth shape or tissue formation or having no phenotypic effect. The pattern of gene expression that emerges corresponds with gene expression patterns that occur in the development of other organ-systems (60).

The dentition has a modular organization (cusps, tooth classes, toothrows) in which suites of morphological characteristics may develop and/or evolve independently from other modules. Such dental modules are expected to share genetic pathways controlling development, and the pleiotropic effects of gene mutations fall to a greater degree within modules than between them (51). For example, the mammalian crown pattern develops through repeated activation of developmental pathways that form a cusp or a cusp-making module (61, 62). Thus, a mutation affecting cusp formation could have repeated effects on the formation of the tooth crown. Furthermore, teeth within a series, such as molars, are expected to develop through repeated morphogenesis of the molar form. Metameric variation occurs when morphogenetic repetition occurs with slight alterations in the development of each unit in the series. In the cases of molars, metameric variations may be seen in the morphological transitions between upper molars (M^1–3^) and have the potential to be taxon-specific. Interestingly, metameric variation was found to be shared and thus highly conserved across apes and humans (63). In a cross-species study of molar size in placental mammals, the growth of the upper molars was found to occur as a unit or module in relation to overall body size. Large-bodied mammals tended to have molars that increase in size from anterior to posterior with the 3rd molar as the largest. In contrast, small or medium-sized species more often show reduction of molar size along the toothrow and are more likely to show reduction or absence of the 3rd molar (64). The toothrow may also be a module that develops and evolves as a unit. For example, upper and lower toothrows may covary with the type of occlusion and jaw movement, such as in caviine rodents (guinea pigs, agoutis). The shape of teeth within the same tooth row shows higher covariation with one another than with opposing teeth, and each series of teeth acts as a functional unit, grinding food through the anterior-posterior movement of the jaw (65).

Stem cells have been identified as an essential component to regeneration of dental tissues as well as a complete tooth. Human dental stem cells with multipotency have been isolated and characterized in order to recreate dental tissue layers, including stem cells from the dental pulp, exfoliated deciduous teeth, apical papilla, periodontal ligament, and dental follicle (66). These cell populations undergo self-renewal and differentiate into multiple cell lineages, forming osseous, odontogenic, adipose, endothelial, and neural-like tissues during *in vitro* and animal studies. A constraint, however, is that human stem cells are limited in their supply for research and clinical applications. As a better alternative, differentiated adult cells have been reprogrammed through treatment with transcription factors like Oct3/4, Sox2, Klf4, and Myc-c to form human induced pluripotent stem cells (iPSCs) (67). IPSCs have high pluripotency and differentiation potential and are a promising source for generating the tissue layers of an artificial tooth (68–70). Researchers are indeed looking to unravel *in vivo* dental differentiation pathways so that induced pluripotent stem cells may be guided into differentiation of tooth-forming structure (71). A valuable tool for periodontal tissue engineering has been the combination of iPSCs with enamel matrix derivative to promote formation of new cementum, alveolar bone, and normal periodontal ligament (72). IPSCs have also shown the potential to differentiate into odontogenic cells, including ameloblasts (73, 74).

Identifying the morphogenetic processes and genetic regulatory pathways that orchestrate tooth development is a pre-requisite to the regeneration of dental tissues, and organoid models have advanced our insight. Organoid models typically include cells that self-assemble into groups suspended in culture media and/or an extracellular matrix scaffold, such as matrigel. The three-dimensional interactions of the cells create *in vitro* conditions like the *in vivo* environment (75). Assembly of human dental pulp cells (hDPSCs) into an organoid that resembles the size of a tooth germ has provided a platform for studying the signaling pathways involved in early stages of human tooth development (76). HDPSCs have also been used to generate organoids with stem cells in the interior and odontoblast-like cells occurring in the outer layer (77). These dentin-pulp-like organoids provide a promising direction for regeneration of the dentin-pulp complex. The formation and uses of organoids as a stepping stone toward tissue regeneration range from tooth germs to other oral and maxillofacial structures such as salivary glands, taste buds, and the temporomandibular joint, as reviewed in Wang and Sun (78).

While there is still a long way to go to develop a completely functional artificial tooth *in vitro*, progress towards this goal has been substantial, as reviewed by Baranova et al. (79). Examples using animal models include a rudimentary tooth germ model generated with recombinant tooth germs implanted into mouse subrenal capsule (80–82), as well as transplantation of a bioengineered mouse tooth germ into the alveolar bone in the lost tooth region (83). Other approaches to whole tooth regeneration have used autologous tooth germ cells to regenerate teeth in dogs, including eruption of the regenerated tooth within the jaw (84).

### 2.4. Dental variation

The literature is replete with surveys of dental variation among populations and species. The most common features to vary differ among taxa but typically include tooth size, supernumerary teeth and/or missing teeth (17, 35, 85–88). In new world marsupials the frequency of observed anomalies reaches as high as 30% in some species (89). When teeth are congenitally absent, this typically occurs at the end of a tooth class; thus, the earlier developing members of the class tend to be more conserved and the later developing members most often missing (90). An unusual case of tooth loss has been noted in a black rat (*Rattus rattus*) in which the incisor dentition was observed to be present, but the molars were absent (91). Another anomaly within rodents has been noted in the deer mouse (*Peromyscus*) in which tooth-like structures have been observed in the diastema, a typically tooth-free zone between the incisors and the molars (92). In the bristly mouse (*Neacomys*), a case of hyperdontia occurred in which a mesial supernumerary tooth occurred anterior to the lower molar (M_1_) and the M_1_ also showed mesial underdevelopment (93).

Tooth shape has also been found to vary among individuals and populations. In some cases, the shape variation includes missing cusps in association with reduced tooth size, such as in the genus *Caluromys* (wooly opossum) (89). Shape changes in association with elongation of the third upper molar were noted between populations and species of vole (*Microtus*) (94). Other variations observed include flipping or rotation of the tooth crown within the dental series (86, 89) or “molarization” in which a premolar takes on the shape of a molar (95). Tooth shape variation may occur when breeding populations become isolated from one another. For example, studies of carnivoran populations separated geographically have shown variations in tooth shape in relation to biogeography such as in arctic fox (37) and raccoon dog. Variations in tooth shape in the raccoon dog (*Nyctereutes*) have been linked with location and multiple species introductions (96).

#### 2.4.1. Carnivorans

The dentitions present among members of the Order Carnivora show an evolutionary reduction of the toothrow and adjustment of the shearing components of the trigonid and crushing regions of the talonid. Some examples are included below in order to illustrate this concept, but a more thorough discussion of the dental forms of these taxa can be found in (7, 97). Carnivora share the loss of the third upper molar (M^3^) (33), placing the remaining teeth closer to the jaw joint, a position of higher bite force. In the upper jaw, the last premolar (P^4^) and in the lower the first molar (M_1_) are specialized into blades, known as carnassials. These specialized teeth are also characteristic of Carnivora and include the lateral flattening of the posterior two cusps on P^4^ and the anterior two cusps on M_1_. As the jaw closes in a scissor-like fashion the blades shear past one another to cut through vertebrate tissue (97). Among extant mammals, marsupials (Tasmanian devil, quoll) have convergently evolved similar shearing blades, however, the blades occur on other teeth (98, 99).

**Figure 4:**
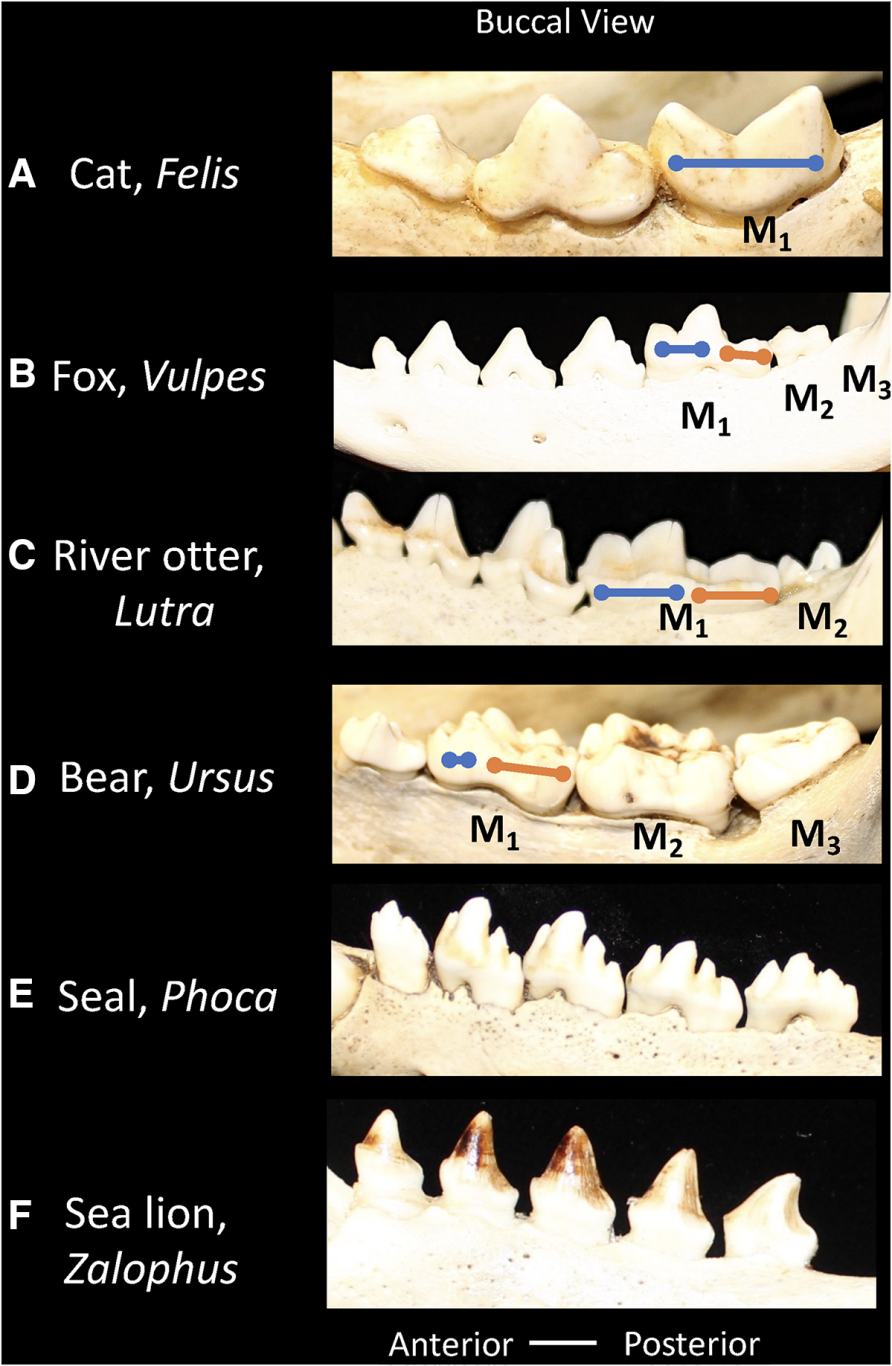
Buccal views of the lower postcanine dentitions of carnivorans showing relative proportions of the trigonid (blue line) and talonid (orange line). (**A**) Cat, *Felis*, the talonid is absent. (**B**) Fox, *Vulpes*, the trigonid and talonid show similar proportions. (**C**) River otter, *Lutra*, the trigonid and talonid show similar proportions. (**D**) Bear, *Ursus*, the trigonid is reduced and the talonid is emphasized. (**E**) Seal, *Phoca*, the trigonid and talonid are absent. (**F**) Sea lion, *Zalophus*, the trigonid and talonid are absent. Dentitions are not shown to scale.

In different Carnivoran subclades, the extent to which the carnassial teeth maintain and/or rely on the bladed morphology differs (Figure 4). For example, in felids (cats) the second and third molars are lost, and the carnassial blades are elongated to maximize the shearing component of the dentition. Thus, in the lower M_1_ the paraconid and protoconid cusps dominate the tooth morphology, and the talonid shows extreme reduction (100). The emphasis of the bladed carnassial morphology corresponds with the high proportion of vertebrate tissue in the diet of most felids. In contrast, many members of the Canidae (dogs) have more balance in the dentition between bladed surfaces and crushing. The carnassial cusps may form a bladed morphology but the talonid on the posterior of M_1_ forms a crushing surface. Furthermore, the canids retain two upper molars and three lower molars and apply these to crushing functions in some species (97). The Mustelidae (ferrets, otters, badgers, wolverine) show diverse modifications of the trigonid and talonid, in association with diet (34). For example, the carnassials of river otters show a balance between bladed and crushing surfaces. Ferrets maintain the carnassial and reduce the talonid crushing surface, whereas badgers' emphasize the crushing functions within the trigonid and talonid, corresponding with the extent of carnivory or omnivory, respectively. Mustelids show further shortening of the toothrow with the upper teeth reduced to only one upper molar, thus ensuring focus of bite forces on the carnassial teeth. In Ursids (bears), most species are generalized omnivores, such as the black and brown bear, the postcanine dentition minimize the carnassial blade and expands crushing surfaces across the dentition. The upper carnassial P^4^ is smaller than the following first and second molars which appear as crenulated crushing surfaces. The lower carnassial includes an expanded carnassial with accessory cusps.

Studies in mutant mice have suggested a potential developmental and evolutionary mechanism for the relative size of shearing vs. crushing surfaces in the dentition. Mice with BMP-7 deficiency have been observed to have pleiotropic effects on tooth size and morphology that are congruent with the evolutionary changes in the relative proportions of M_2_/M_1_ as well as the trigonid and talonid in Carnivora. Evolutionary modifications of BMP-7 signaling during tooth development have been proposed as a mechanism for generating the phenotypic changes in the dentition that correspond with dental adaptations to different diets (101).

The pinnipedia, including phocids (seals), otariids (sea lions) and odobenids (walruses) belong to the Order Carnivora, but have lost the primitive dental features that characterize most terrestrial carnivorans (Figure 4). As descendants of a putative weasel-like ancestor, their predecessors possessed a carnassial tooth specialization. This carnassial specialization has been secondarily lost however in favor of a more simplified tooth morphology. The tooth row tends towards homodonty in which each tooth in a series is similar in form rather than differing in shape in correspondence with tooth classes (heterodonty). Pinnipeds feed on aquatic prey, and in general the dentition does not function in chewing, but in capturing prey and swallowing it whole. The absence of a precise occlusion, or the matching of tooth surfaces that coordinate in food breakdown, has led to the supposition that the simplified teeth in the pinniped dentition may show more variation.

The hypothesis that species with precisely occluding carnassial teeth may be under higher stabilizing selection relative to more homodont species has mixed support. Researchers have assessed tooth size variability as an indicator of the potential for tooth variation to interfere with occlusion. Meiri et al. observed a higher correlation between the size of carnassial teeth in carnivores feeding mainly on vertebrates compared with more insectivorous or frugivorous species, suggesting that species that required precisely occluding carnassial teeth to breakdown tough vertebrate tissue could be under high stabilizing selection (102). Furthermore, molar size variation in Newfoundland black bears, omnivorous in their diet and showing simplified carnassials, has been observed to be intermediate between pinnipeds and carnivorans that retain a carnassial specialization, such as canids (103). Greater variation in tooth size in ringed and harp seals has been reported relative to some carnivorans, such as fox, in which the carnassial food-processing is maintained (104, 105). Wolsan et al. observed that size variation differed considerably both within and among species of pinniped but did not find evidence that the evolutionary simplification of tooth shape contributed to greater variation. This wide spectrum of levels of size variation in pinnipeds did indeed include some high levels, such as in otariids (fur seals and sea lions) but also included the low variation observed in terrestrial carnivorans (106). Furthermore, evaluation of tooth size variation across several families of terrestrial carnivores that vary in their reliance on precise occlusion did not support the hypothesis that poorly occluding teeth were more variable than carnassials (85).

#### 2.4.2. Suoidea

**Figure 5:**
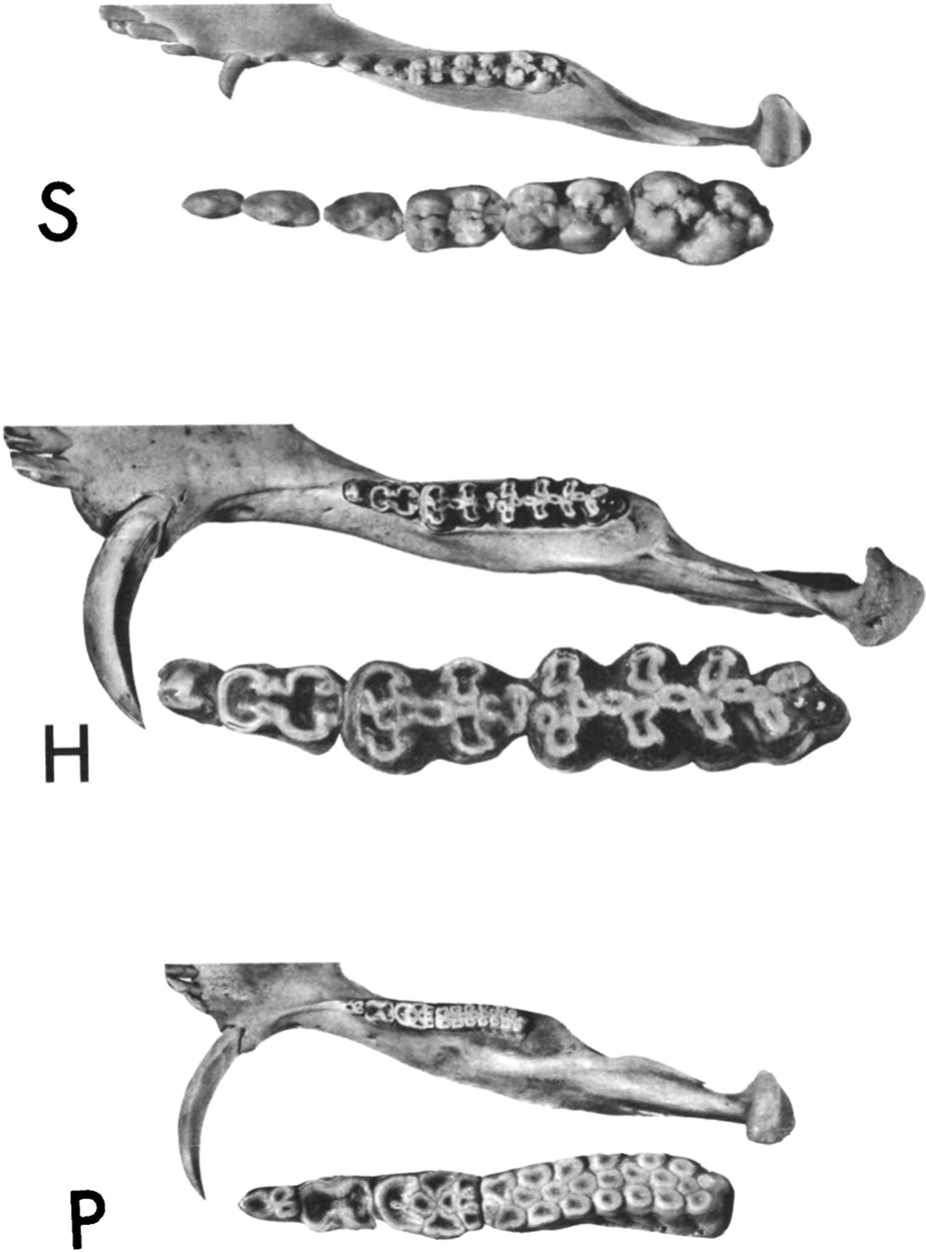
Occlusal views of the left mandibular halves of the pig, *Sus* (S), the giant forest hog, *hylochoerus* (H), and warthog, *phacochoerus* (P). The cheek teeth of each are enlarged and inset. Reprinted with permission from the publisher, Oxford University Press.

**Figure 6:**
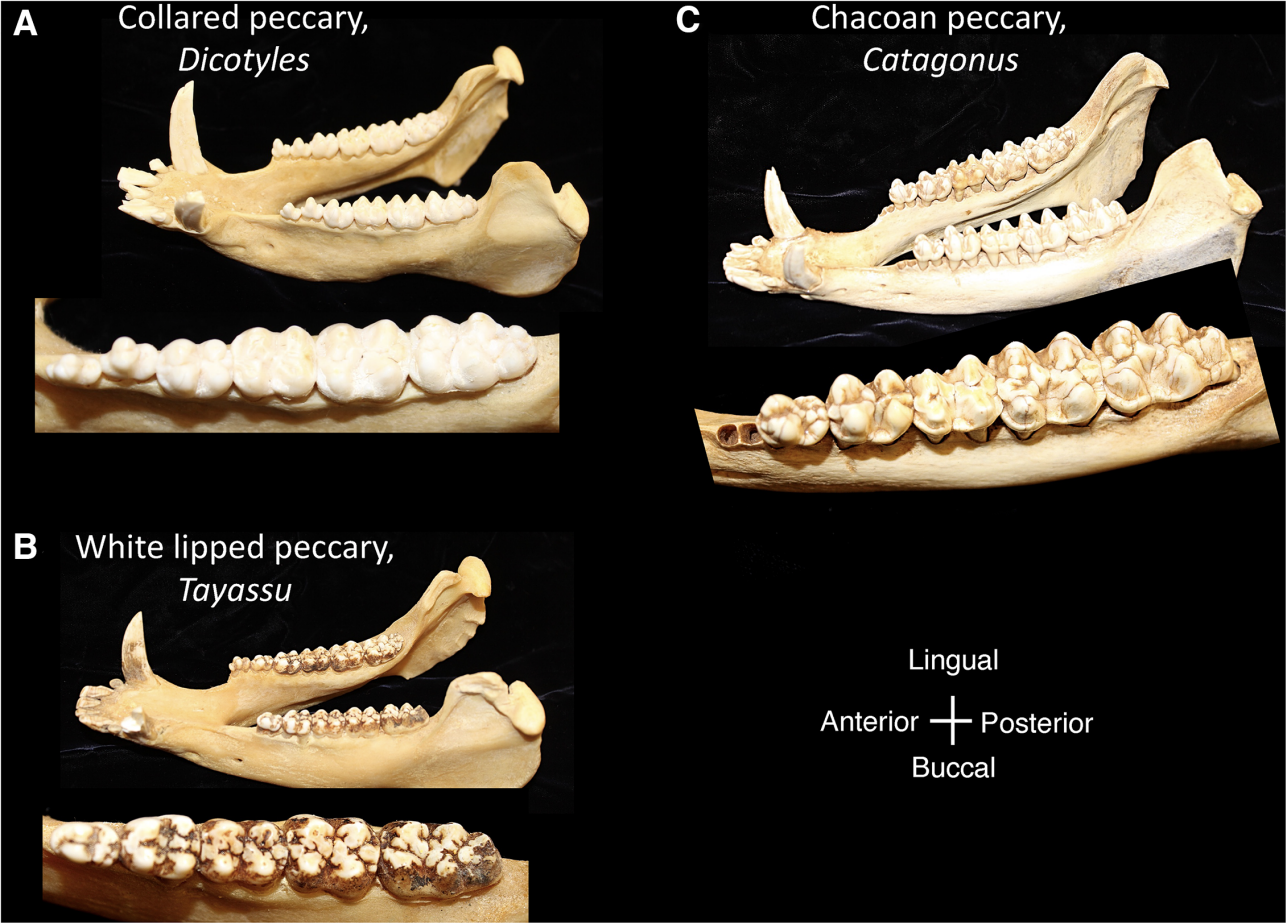
Mandibles and postcanine mandibular tooth rows of the tayassuidae, (**A**) collared peccary, *Dicotyles*, (**B**) white lipped peccary, *Tayassu*, (**C**) chacoan peccary, *Catagonus*. Dentitions are not shown to scale.

Within the Order Artiodactyla, the dentitions of Suoidea show morphological changes from the primitive mammalian dentition to increase surface area for chewing. The superfamily Suoidea consists of the Suidae (pigs, boar, hogs) and Tayassuidae (peccaries) and among artiodactyls display the most primitive traits. These species are omnivorous and most include low-crowned teeth and rounded cusps (bunodont), although the warthog and giant forest hog include higher dental pillars (Figure 5) (107). Suid molars include four main cusps, including the hypocone that has evolved in parallel in several mammalian lineages in association with herbivory (108). Relative to the three-cusped, tribosphenic molar primitive to mammals, this “quadritubercular molar” includes a greater surface area for processing plants in the diet. In the lower molar, the protoconid and hypoconid occur buccally and metaconid and entoconid lingually (Figure 2); the paraconid cusp is absent and regarded as fused to the metaconid (109). Suid molars are also distinctive in the presence of three furrows surrounding each of the major cusps (110). The basined valleys between cusps often include additional cusplets. The chewing surface area of the postcanine teeth is enlarged with the “molarization” of the fourth premolar, a tendency for the tooth shape to resemble the first molar (7). The third lower molar is expansive and shows a large cuspidate talonid in addition to the quadritubercular anterior of the tooth (111). The peccary dentition is like that of pigs, with premolars increasing in size and complexity with the last premolar (P3) appearing molariform (Figure 6). The molars are also bunodont and quadritubercular, however, the occlusal surface is simpler than in pigs and appears bilophodont (7). The Chacoan peccary (*Catagonus*) differs from other peccaries (*Tayassu*) in the presence of larger molars that include a lophodont pattern or the occurrence of ridges rather than cusps. The occlusal area in the Chacoan peccary is larger than that of other peccaries, but closer in size to pigs *Sus* (107).

Suoids share the characteristic of a functional postcanine tooth shape that is wear-derived. In pigs, wear facets develop on the mesial and distal cusp surfaces and unite cusps into transverse lophs (112). The enamel crumbles easily when load is applied, and during continued wear, dentin pools are surrounded by infolding enamel rings. More extensive wear reduces the cuspal contours and the crown surface appears flat. The complexity of the tooth crown ensures that, despite the degradation of the enamel, a range of dental features are available to concentrate stresses in food during chewing (113). Within the Suoidea taxa are mainly omnivorous or herbivorous, and the wear-derived dental morphology has permitted consumption of these varying food types.

Geometric morphometrics have been used to capture the curvature differences between the outlines of premolars and molars and to relate phenotypic differences to the biogeography of wild boar as well as to different stages in the domestication process (114, 115) Two-dimensional (2D) tooth outlines are often used for comparison of tooth shape among Suoids because the wear on the occlusal surface makes it difficult to use occlusal landmarks in morphometric analyses (111). The 2D outlines capture the position of the main and supernumerary cusps and differences in tooth form were distinguishable between wild and domestic pig populations. For example, the molars from domestic animals were smaller in size than wild boar and the molars from wild boar populations showed variation in accordance with biogeography (115).

#### 2.4.3. Primates

**Figure 7:**
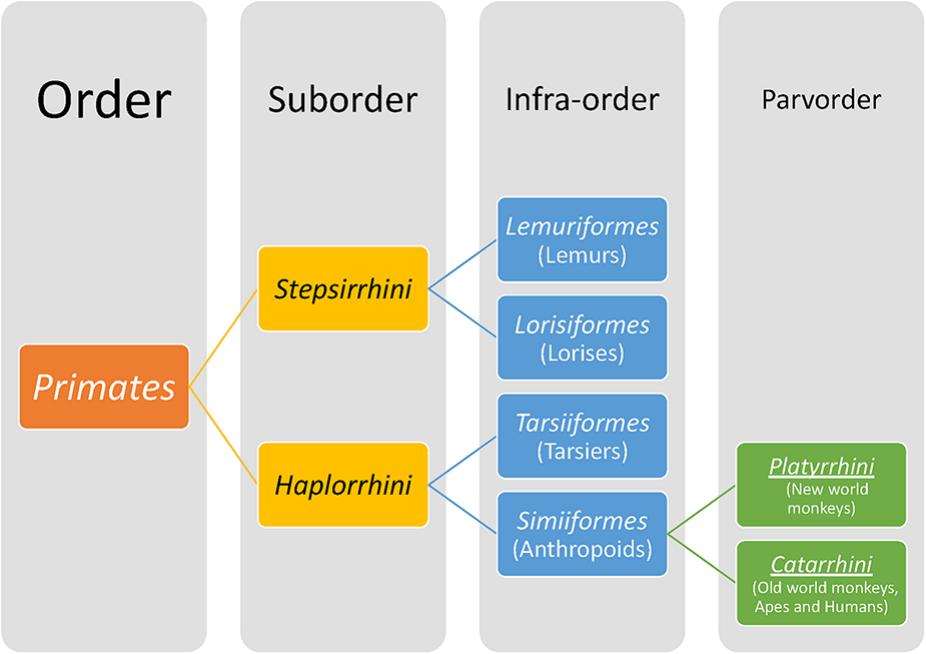
A brief classification of primates.

Members of the Order Primates (Figure 7) have distinctive features from other mammals that include a relatively large brain with cortical folding, prehensile (grasping) hands and feet, opposable thumbs and/or great toes, flattened nails on digits instead of claws, acute vision and prolonged postnatal dependency of offspring. The Order Primates includes lemurs, lorises, tarsiers and anthropoids (monkeys, apes and humans). Hominoids belong to the superfamily Hominoidae and are distinguished from the rest of the primates by their absence of tails and flexible shoulder joints enabling arboreal locomotion by swinging from arm to arm (brachiation). Hominoids as a term refers to Hominins (current humans and their extinct ancestors of genera *Homo*, *Autralopithecus*, *Paranthropus* and *Ardipithecus*) and apes (gorilla, chimpanzees, orangutan, bonobos, gibbons). The dentition of primates reflects an evolutionary mammalian trend of an increase in the number of cusps and decrease in number of teeth. All primates have two incisors, one canine and three molars but varying number of premolars. No living primate has four premolars; three premolars per quadrant/toothrow are found in lemurs and new world monkeys (found in Central and South America) whereas in Catarrhines, i.e., old world monkeys (natives of Asia and Africa), apes and humans, the premolar number has dropped to two (7, 116). The posterior-most premolars undergo molarization by evolving to have either one or two extra cusps as opposed to primitive premolars that are unicuspid and uniform in shape. Similarly, the trend in molars has been to upgrade the primitive three-cusp molar to a four- or five-cusped molar.

The tooth morphology of primates is adapted to eat plants and mixed food. As in other mammals, the incisors bite off pieces of food and the premolars and molars grind them. Tooth size in extant primates is found to be correlated with their dietary regimes. Frugivorous primates have relatively larger incisors to be able to dehusk fruits and seeds, and their molars are bunodont. Compared to frugivores, leaf-eating primates tend to have smaller incisors and molars with relatively higher cusps, sharp shearing crests and larger crushing surfaces that enable longer chewing times for processing the tough, low-energy-value leaves (7, 117). In contrast, insect-eating primates have smaller molars with sharp cusps to puncture insect exoskeleton. Non-dietary functions of tooth size are related to grooming and social functions in primates. The canine honing complex is a functional complex where a long, conical, projecting upper canine is continually sharpened by occlusion against the lower second or third premolar, a dental trait that is nearly ubiquitous in both extant and fossil anthropoid primates except humans (118). Kronfeld (119) suggests that the projecting canines in non-human primates act as formidable weapons especially in the males in which they are larger and stronger compared to females. Investigations in the late 1970s of relative canine size in relation to social organization have found that sexual dimorphism in canine size was greatest in taxa in which intragroup selection and predator pressures are significant influences (117). However, a more recent study refutes the argument that the honing complex is selectively important only in males as it is found equally in both male and female non-human anthropoids. Additionally, the study found no evidence for differences in either among- or within-species phenotypic covariation between male and female anthropoids (118).

The dentition of modern-day humans is most similar to anthropoid apes that includes the chimpanzee, gibbon, gorilla, and orangutan, with important differences (120). The first signs of modern human dentition, presence of 2-1-2-3 dental formula and loss of the canine honing complex, were found in Miocene apes or hominoids (22.5–5 mya) (121). The evolution of hominoids from the Miocene to the Pliocene period (∼5–2.5 mya) saw a gradual reduction in canine length, enamel thickness of molars and cuspal heights. The transition from genera *Paranthropus* and *Australopithecus* during the Plio-Pleistocene period (from around 5 mya to 12 kya) to *Homo* resulted in reduction in facial prognathism, rectangular crown shape, a more parabolic archform as opposed to an earlier rectangular one, reduction in postcanine crown sizes, a characteristically large M1 compared to posterior molars, more variable M3 cusp patterns and an eventual reduction in root size. Overarchingly, the hominins showed reduction in both crown and root sizes, with some evidence existing that the former preceded the latter (116). Changes in nature of foods consumed (like ease of fracture, toughness, abrasiveness, amount, etc.) might have reduced the functional loads on dental crowns causing a subsequent reduction in root morphology and size. This deficiency of human teeth both in number and structure has been attributed to an advanced development of the brain, leading to the use of weapons and tools to cook food, thus reducing the relative importance of teeth throughout evolution. The differences between human dentition and other closely related anthropoids are shown in Table 1 [adapted from Kronfeld (119)].

**Table 1:** Differences between human and non-human anthropoid primates dentitions [adapted from Kronfeld (119)].

	Non-human anthropoid primates (monkeys, apes)	Humans
1	The incisors are inclined forward	The incisors are nearly vertical in both jaws.
2	The dental arch is long, narrow, and square.	The dental arch is short and rounded.
3	A space (diastema) exists between upper lateral incisor and upper cuspid to accommodate the crown of the projecting lower cuspid.	There are no spaces between the individual teeth of either jaw; all teeth are in contact with each other
4	The crowns of the canines are much longer and stronger than those of the incisors and premolars. The canines protrude above the other teeth.	The crowns of the canines are the same length as those of the incisors and premolars. All teeth are the same level
5	The upper premolars have three roots, the lower premolars two roots.	The upper premolars have two roots the lower premolars one root
6	The molars increase in size from the first to the third molar.	The molars decrease in size from the first to the third molar.

In addition to the increase in morphological complexity, mammals evolutionarily have also moved towards reducing the number of times the dentition is replaced over the organism's lifetime. Unlike most fish and reptiles that have a permanent dental lamina that replaces teeth throughout the lifetime of the animal (polyphyodonty), most mammals including primates, pigs and ferrets are diphyodont whereas rodents are monophyodont. The diphyodont dentition in humans consists of two sets of teeth—the deciduous dentition and its replacement, the permanent dentition. Premolars are absent in human deciduous dentition, which has the dental formula of two incisors, one canine and two molars. Interestingly, the succedaneous teeth for deciduous molars are not permanent molars but rather premolars. The permanent human dentition consists of two incisors, one canine, two premolars and three molars. It is postulated that the human dentition is a result of evolutionary suppression of the distal-most third incisor and the first and second premolars usually found in the mammalian dentition. Hence, the first and second premolars (P1, P2) found in humans are considered homologous to the third and fourth premolars (P3, P4) in other mammals. Ontologically, Hovorakova et al. demonstrate the existence of a transient gap in human embryos between the developing deciduous canine and deciduous first molar as evidence of the evolutionarily missing deciduous mammalian premolars in humans (122). Many of these intra- and inter-species variations in hominoid dental characteristics have been used as the basis of more comprehensive investigations on human evolution, dietary habits and population history. For instance, increased tooth sharpness in hominins compared to extant great apes indicates a diet higher in plant or animal-based fiber intake [36]. The study of mammalian dentition in general and of primate dentition in particular thus continues to fuel development of hypotheses and experimentation in the quest for precise odontogenic mechanisms that could one day facilitate *in vitro* odontogenesis for clinical transplantation in patients.

## 3. Discussion

An opportunity for evolution to inform tooth regeneration has been observed in the multiple rows of replacement teeth that occur in many vertebrates including fish and reptiles (123). As discussed in Section 2.4.3, most mammals, including primates, are limited to two sets of teeth. The dental lamina includes the ectodermal precursor tissue to developing teeth and retains the capacity to generate new teeth through expression of the stem cell factor Sox2 in reptiles and mammals (124–126). The expression of Sox2 in epithelial tissue near the developing replacement teeth in cichlid fish suggests that Sox2 maintains the tooth forming ability of the epithelium across vertebrates (123). Because mammals share a common ancestor with other vertebrates, the regulatory pathways in polyphyodont vertebrate dentitions where teeth are constantly replaced may provide input for dental regeneration. Unraveling control mechanisms that maintain the dental lamina permanently in polyphyodonts for lifelong tooth generation can provide tissue engineers with critical cues to intiate regeneration of new sets of teeth in humans beyond the diphyodont dentition. Conversely, mechanisms that could prevent degradation of dental lamina after permanent dentition eruption that happens in diphyodonts and induce odontogenesis anew could be another approach.

Studying the evolution of mammalian dentitions provides a historical record of how odontogenic pathways arrange and build the various modular parts of a developing tooth and dentition. Evolutionary studies have been primarily built on the analyses of teeth as they make up a disproportionate number of fossils discovered. The solid encasing provided by enamel, the hardest substance in the body with 96% mineralized content, allows teeth to be preserved better than any other part of the skeleton. The relatively unchanged enamel, which does not remodel after tooth eruption unlike bone or other dental tissues, acts as a perfect time capsule. Although cellular and molecular interactions may not fossilize, the developmental processes that generated tooth shapes can often be inferred from the fossilized structure through comparison with living mammals. Thus, evolutionary variations in dental patterns can provide researchers insights into the developmental mechanisms that produce dental patterns.

Cross-species comparisons and an interdisciplinary perspective are essential in identifying the drivers of tooth shape variation, evolutionary change, and/or the developmental pathways for tooth regeneration. Assessing tooth shape change in the context of phylogenies that incorporate both molecular evidence and the fossil record makes it possible to tease apart the relative contribution of phylogenetic history and functional adaptation to the shape differences. This approach may also demonstrate the evolutionary timing of the shape change among taxa relative to the more primitive morphology. Developmental and molecular biologists have illuminated the genetic and ontogenetic underpinnings of tooth shape differences. Viewing these differences in a phylogenetic context across a broad range of taxa could offer new insights into the evolution of dental diversity and approaches to regeneration of tooth shape. For example, comparative study of mammalian dental development has defined the common role of homeobox gene expression in defining tooth classes within the developing jaws (42). Comparison of the ranges of homeobox gene expression can be linked to variations in tooth class number between species (50). The evolutionary conservation of homeobox gene expression suggests that this patterning may be recreated in regenerative approaches to specify tooth class differences (127). Mutant mouse models are commonly used in the assessment of the genetic effects on morphology; however, a comparison of developmental pathways in mice with a broader range of species could also be a powerful tool in illuminating the developmental differences that generate tooth shape differences. This may be unrealistic for many mammalian species, due to ethical considerations and/or practicalities in accessing ontogenetic stages for study; however, the animal models commonly used within biomedical research do offer taxonomic diversity (rat, rabbit, ferret, dog, pig). Indeed, comparative study across a broad range of taxa has enabled the identification of common signaling pathways that are used repeatedly in dental development (45). Comparative study could also pinpoint the factors that direct the formation of species-specific tooth shapes using a common array of signaling pathways.

Tooth shape and cusp homology are deeply rooted within vertebrate evolutionary history and odontogenic pathways and control mechanisms are also expected to be highly conserved within related taxa. As such, the evolutionary conservation and commonality of developmental pathways offer a potential developmental “roadmap” to the regeneration of dental tissue in a laboratory setting. Although signaling pathways are used repeatedly in tooth development, the spatial and temporal use of these shared pathways has undergone evolutionary change in order to generate mammalian tooth shape diversity (45). The capacity for tissue engineers to similarly alter the use of these pathways could be applied to the regeneration of desired tooth shapes for clinical use. The potential effects of the modification of temporal and spatial activation of cusps are demonstrated in computational analysis of virtual inner enamel epithelium in 17 living and fossil hominoid species (128). The interplay between the timing and spacing of enamel knot initiation and the duration of crown growth before mineralization determines molar shape in hominoids. A blueprint for crown morphogenesis can be seen in the enamel knot's repeated activation and silencing of diffusible signaling molecules. Current regenerative approaches have yet to regenerate tooth shape through enamel knot signaling, however, *in vitro* and *in vivo* methods have achieved crown shape using biodegradable scaffolds in a preformed shape. *In vitro* and *in vivo* studies have demonstrated the capacity for stem cells of different types to be differentiated into dental tissues akin to odontoblasts and ameloblasts using specific combinations of signaling molecules (79, 127). In future tooth regeneration approaches it may be possible to regenerate enamel knots and control cusp formation through regulation of enamel knot activity. The capacity for altering tooth shape with increasing the number of iterations of cusp formation is observable in comparing mice and vole gene network pathways superimposed on their dental topography (129). Such minor tweaks of morphogenesis that generate teeth of various shapes and sizes demonstrate how tooth shape and cusp form can be modified while developing an artificial tooth *in vitro*. To harness these processes for tooth regeneration, further studies are needed to link genetic and developmental differences to changes in tooth shape.

The appearance of dental variation between individuals and populations also has the potential to show us deviancy in the developmental program and the underpinnings of human dental anomalies. Many dental variations are likely to correspond with minor changes in the developmental program, such as signaling changes that produce more extensive growth of a dental feature and increase cusp or tooth size. Alternatively, the truncation of development may result in changes in size, presence, absence, or number of tooth cusps or teeth themselves (130). Such edits to developmental pathways not only form the basis for tooth shape variation within species but are likely to have been co-opted in the evolution of taxon-specific tooth shape differences. Other mechanisms that produce more abrupt or “discontinuous” variations in tooth morphology, however, are also likely to be afoot. In 1894 Bateson noted a common assumption regarding evolution, namely that variation occurs in a continuous series and that natural selection optimizes a morphology for functional performance. Bateson found that this assumption did not fit with the evidence of the natural world. He instead noted the sudden and discontinuous nature of variation, such that morphological variation occurred *de novo* without similar variants in form. Rather than being shaped under the guiding influence of natural selection, he proposed that species differences could be a result of the processes creating “discontinuous” variation (5). Although Bateson ascribed this discontinuous variation to the “intrinsic nature of organisms themselves”, our contemporary understanding of tooth development allows more definition to this hypothesis. For example, the modular development of the dentition creates the opportunity for pleiotropic effects, whereby simple mutations may have far-reaching and seemingly unrelated effects on the dentition. Furthermore, mutations that occur within a developmental module have the potential to be amplified during the repeated expression of the module. Such mechanisms may underlie the anomalous tooth shapes that occur in the human dentition. Developing a greater understanding of the dentition's modular development and pleiotropic effects of mutations could improve our understanding of developmental abnormalities in the dentition and in turn lead to preventative therapies.

In sum, mammalian dental evolution and existing dental diversity demonstrates the variety of dentitions and tooth shapes derived from a common ancestor in the Late Jurassic. Evolutionary processes have modified the developmental program for production of species-specific dentitions that function in a range of dietary categories. That dentitions maintain homology with one another at the level of the tooth and tooth cusp underscores the conservation of the cellular and molecular events involved in their formation. It follows that given the right conditions, autologous stem cells or hIPCSs could be guided into forming mature dental tissues. Continued study of the developmental events that generate mammalian dentitions and comparative study of the mechanisms that result in diverse morphologies will continue to point the way toward regeneration of the dentition. By mimicking tooth development it could be possible to regenerate various dental tissues, guide cusp development, direct crown morphogenesis and eventually generate a fully functional, laboratory-created artificial tooth for replacement. Combining our insight from the evolutionarily conserved developmental pathways that generate diverse mammalian tooth shapes with advanced research methods including iPSCs and organoids offers a promising future for whole tooth regeneration.
